# Hepatitis B and C infections among Japanese dental health workers: Insights from vaccination rates and screening results in the Oita prefecture

**DOI:** 10.1002/cre2.871

**Published:** 2024-03-20

**Authors:** Yumiko Nagao, Tetsuya Kimura, Kiyohide Tomooka, Haruhiko Wakita

**Affiliations:** ^1^ Department of Public Health, Graduate School of Medicine Juntendo University Tokyo Japan; ^2^ Liver Center Saga University Hospital Saga Japan; ^3^ Oita Dental Association Oita Japan

**Keywords:** dental healthcare workers, hepatitis B vaccination, hepatitis B virus, hepatitis C virus

## Abstract

**Objective:**

This study examined the hepatitis B virus (HBV) and hepatitis C virus (HCV) infection rates and vaccination rates for hepatitis B (HB) among dental healthcare workers (DHCWs) in the Oita prefecture, Japan.

**Methods:**

Hepatitis virus testing was conducted on 1920 participants (486 dentists and 1434 dental staff). Anonymous data on age, gender, occupation, hepatitis B surface antigen (HBsAg), antibodies to hepatitis B surface antigen (anti‐HBs), antibodies to HCV (anti‐HCV), history of HB vaccination, and antiviral treatment for individuals with positive anti‐HCV were collected.

**Results:**

The positivity rates for HBsAg, anti‐HBs, and anti‐HCV were 0.5%, 39.7%, and 0.6%, respectively. Dentists had significantly higher rates of anti‐HBs positivity (53.9% vs. 34.9%; *p* < .0001) and anti‐HCV positivity (1.4% vs. 0.3%; *p* = .0080) compared to dental staff. The vaccination and non‐vaccination rates among 1395 with a known HB vaccination history were 59.1% and 40.9%, respectively. Dentists had a significantly higher HB vaccine vaccination rate than the dental staff (73.6% vs. 54.0%; *p* < .0001). Those in the vaccination group were younger (*p* < .0001), had a higher proportion of males (*p* = .0022) and dentists (*p* < .0001), a lower HBsAg positivity rate (*p* < .0097), and a higher anti‐HBs positivity rate (*p* < .0001) compared to those in the non‐vaccination group. The positivity rate of HBsAg and anti‐HBs in the unvaccinated group increased with age, with HBsAg positivity reaching 3.8% in the 70s and anti‐HBs positivity reaching 40.4% in the 70s and 66.7% in the 80s.

**Conclusions:**

This study highlights the need to raise awareness about hepatitis prevention vaccination, particularly among dental staff, due to differences in HB vaccination rates across occupations. In particular, they indicated that elderly DHCWs may be more vulnerable to HBV infection. Regular monitoring of the vaccination rate and infection risk is crucial.

## 1 INTRODUCTION

Dental healthcare workers (DHCWs) must focus on infection control measures and occupational infection prevention due to frequent exposure to blood and body fluids (Sebastiani et al., 2017). Infections by bloodborne pathogens such as hepatitis B virus (HBV) and hepatitis C virus (HCV) are significant concerns. HBV is highly infectious; therefore, the vaccination of DHCWs is crucial to prevent occupational infections (Bromberg & Brizuela, 2023). In Japan, there are an estimated 1.91−2.49 million HBV‐ and HCV‐infected individuals, with many unaware of their infection status (Tanaka et al., 2022). Therefore, healthcare workers need to rigorously implement infection control measures based on standard precautions and receive hepatitis B (HB) vaccination. The etiology of hepatocellular carcinoma (HCC) varies by region, with HCV infection being the primary cause in Japan, while HBV infection is a major factor in South Asia and Africa (Llovet et al., 2021).

Efforts to prevent HBV infection in Japan include “Hepatitis B Mother‐to‐Child Infection Prevention Project,” which began in 1986, and the universal vaccination, which began in 2016. The project for prevention of mother‐to‐child transmission of HB offers HBV testing and vaccination to pregnant women to prevent mother‐to‐child transmission. In addition, a routine HB vaccination program was initiated for infants at birth. This has led to the implementation of comprehensive preventive measures in Japan, not only for mother‐to‐child transmission. Regarding generational differences in HBV infection, the majority of chronic HB patients in Japan are elderly (Yotsuyanagi et al., 2022). On the other hand, the introduction of universal vaccination is expected to reduce the risk of infection among the younger generation and eliminate new infections in Japan (Tanaka et al., 2019).

In 2009, the Japanese Society for Infection Prevention and Control established the “Vaccine Guidelines for Healthcare Workers,” which states that all healthcare professionals who may come in contact with patients, their blood, or environmental surfaces contaminated with blood should receive HB vaccination (Mikamo et al., 2020; Okabe et al., 2009). However, in one of our previous studies, we reported a high HBV infection rate and a low HBV vaccination rate among DHCWs in the Fukuoka prefecture in Japan (Nagao et al., 2008). Additionally, Tada et al. (2014) reported that infection control practice was not widely practiced in dentistry in Japan. Similar findings were observed in another recent study conducted by our group, wherein dentists did not always practice safe medicine (Nagao, 2018). Subsequently, proactive measures were taken to address this issue by introducing a regular hepatitis testing program and conducting hepatitis education activities in the Oita Dental Association, which was a pioneering nationwide effort (Nagao et al., 2021, 2022). Specifically, 1834 DHCWs underwent hepatitis virus testing during regular health check‐ups in 2018. The results showed significantly higher HBV and HCV infection rates among the participants than in the first‐time blood donors (*n* = 2,727,727) in Japan (Nagao et al., 2021). The positivity rates for hepatitis B surface antigen (HBsAg) and antibodies to HCV (anti‐HCV) increased with age; the age group of 50−70 years had the highest infection rate (positivity rate of 1.7%−2.2%).

In 2021, an educational program on hepatitis was conducted for 2197 DHCWs in the Oita prefecture (Nagao et al., 2022). The survey revealed that 61.6% of participants had experienced percutaneous injuries and lacked sufficient understanding of the appropriate response measures before reading the educational material. However, 99.5% of respondents rated the educational material as useful, indicating its potential to enhance the motivation for hepatitis testing.

Based on these backgrounds, the aim of this study was to investigate the relationship between hepatitis virus infection and the HB vaccination status by conducting a second hepatitis screening in 2022, 4 years after the initial screening in 2018, among DHCWs in the Oita prefecture.

## 2 MATERIALS AND METHODS

### 2.1 Subjects

Between April 2022 and March 2023, a total of 1920 DHCWs (including dentists and dental staff) from the Dental National Health Insurance Society in the Oita prefecture underwent hepatitis virus testing during their regular health check‐ups at contracted medical facilities. During the testing, they were asked to respond to a questionnaire regarding their vaccination history for HB and antiviral therapy for hepatitis C. Written consent to provide their health examination results to third parties for health management and effective utilization purposes was obtained from each participant. The ages of the 1920 participants (487 males and 1433 females) ranged from 19 to 81 years, with an average age and standard deviation of 45.2 ± 15.1 years. The participants comprised 486 dentists and 1434 dental staff members.

### 2.2 Study design

The results of the health examinations were individually notified to each participant via sealed letters from the medical testing center. Anonymized data provided by the Dental National Health Insurance Society in the Oita prefecture included age, gender, occupation (dentist or dental staff), HBsAg, hepatitis B surface antibody (anti‐HBs), anti‐HCV antibody, vaccination history for HB, and history of antiviral treatment for hepatitis C among individuals positive for anti‐HCV. The data were collected and analyzed for the study.

### 2.3 Assays for HBsAg, anti‐HBs, and anti‐HCV

The serum HBsAg level and anti‐HBs titer were measured using commercially available chemiluminescent immunoassay kits (Architect HBsAg QT Abbott and Architect HBs Antibody kit Orsab Abbott, respectively; Abbott Japan Co. Ltd.) according to the manufacturer's instructions (cut‐off index [COI], <0.05 IU/mL and 10 mIU/mL, respectively).

Serum anti‐HCV was assessed using an automated chemiluminescent enzyme immunoassay kit (Lumipulse II HCV; Fujirebio Inc.). A COI of >1.0 was considered indicative of a positive result for anti‐HCV antibodies, whereas values <1.0 were considered negative.

### 2.4 Survey of HB vaccination and history of antiviral therapy for hepatitis C

The subjects were required to respond to a questionnaire regarding their history of HB vaccination at the time of blood collection; the response options included “vaccinated,” “never vaccinated,” and “unknown.” Additionally, information about antiviral therapy for hepatitis C, using the “yes,” “no,” and “unknown” options, was collected.

### 2.5 Statistical analysis

Data are expressed as the mean ± standard deviation. Differences between the two groups were analyzed using Wilcoxon's signed rank and Fisher's exact tests. All statistical analyses were performed using JMP version 13 (SAS Institute Inc.). A *p*‐value of less than 0.05 was considered statistically significant.

### 2.6 Ethics statement

The study protocol was approved by the Ethics Committee of the Oita Dental Association (reference number: 2020‐1) in accordance with the Declaration of Helsinki. Written informed consent regarding the submission of the results of the medical examination to a third party for health management and effective use was obtained from each subject.

## 3 RESULTS

### 3.1 Positivity rates of HBsAg, anti‐HBs, and anti‐HCV, and the HB vaccination rate

The positivity rates of HBsAg, anti‐HBs, and anti‐HCV among all subjects were 0.5% (10/1920), 39.7% (763/1920), and 0.6% (11/1920), respectively (Table 1). Regarding the HB vaccination coverage, 42.9% of the subjects were vaccinated, 29.7% had never been vaccinated, 8.7% did not know their vaccination status, and 18.7% did not respond.

**Table 1:** Characteristics of the 1920 subjects.

Characteristics		Total	Dentists	Dental staff	*p* Value
Occupation		1920	486 (25.3%)	1434 (74.7%)	<.0001
Age, mean ± standard deviation, years		45.2 ± 15.1	56.1 ± 13.1	41.5 ± 13.9	<.0001
Sex	Male	487 (25.4%)	407 (83.7%)	80 (5.6%)	<.0001
	Female	1433 (74.6%）	79 (16.3%)	1354 (94.4%)	
Age, years, *n* (%)	19	2 (0.1%)	0 (0%)	2 (0.1%)	NS
	20−29	406 (21.1%)	3 (0.6%)	403 (28.1%)	<.0001
	30−39	318 (16.6%)	61 (12.6%)	257 (17.9%)	.0058
	40−49	425 (22.1%)	104 (21.4%)	321 (22.4%)	NS
	50−59	363 (18.9%)	93 (19.1%)	270 (18.8%)	NS
	60−69	293 (15.3%)	135 (27.8%)	158 (11.0%)	<.0001
	70−79	109 (5.7%)	86 (17.7%)	23 (1.6%)	<.0001
	80−89	4 (0.2%)	4 (0.8%)	0 (0%)	.0041
HBsAg, *n* (%)	Positive	10 (0.5%)	4 (0.8%)	6 (0.4%)	NS
	Negative	1910 (99.5%)	482 (99.2%)	1428 (99.6%)	
Anti‐HBs, *n* (%)	Positive	763 (39.7%)	262 (53.9%)	501 (34.9%)	<.0001
	Negative	1157 (60.3%)	224 (46.1%)	933 (65.1%)	
Anti‐HCV, *n* (%)	Positive	11 (0.6%)	7 (1.4%)	4 (0.3%)	.0080
	Negative	1909 (99.4%)	479 (98.6%)	1430 (99.7%)	
HB vaccination	Vaccinated	824 (42.9%)	267 (54.9%)	557 (38.8%)	<.0001
	Never vaccinated	571 (29.7%)	96 (19.8%)	475 (33.1%)	
	Unknown	167 (8.7%)	32 (6.6%)	135 (9.4%)	
	Information not provided	358 (18.7%)	91 (18.7%)	267 (18.6%)	

*Note*: Differences between the two groups were analyzed using Wilcoxon signed‐rank test and Fisher's exact test.

Abbreviations: anti‐HB, antibody to HBsAg; HB, hepatitis B; HBsAg, hepatitis B surface antigen; HCV, hepatitis C virus; NS, not significant.

Significant differences in age, gender, age group, anti‐HB positivity rate, anti‐HCV positivity rate, and HB vaccination rate were observed between the dentists and dental staff. The dentists were older (56.1 ± 13.1 vs. 41.5 ± 13.9; *p* < .0001), predominantly males (83.7% vs. 5.6%; *p* < .0001), and had significantly higher rates of anti‐HBs positivity (53.9% vs. 34.9%; *p* < .0001), anti‐HCV positivity (1.4% vs. 0.3%; *p* = .0080), and HB vaccination (54.9% vs. 38.8%; *p* < .0001) than the dental staff. No significant difference in the HBsAg positivity rate was observed between the two groups.

### 3.2 Occupational differences in vaccination coverage among the 1395 subjects with confirmed HB vaccination status

The vaccination rate in 1395 subjects whose HB vaccination status was confirmed, after excluding the 167 who answered “do not know” and 358 who did not provide information about their HB vaccination history, was 59.1% (824/1395), and the unvaccinated rate was 40.9% (571/1395; Table 2). Dentists had significantly higher HB vaccination coverage than the dental staff (73.6% vs. 54.0%; *p* < .0001).

**Table 2:** Comparison of the vaccination rates among 1395 subjects with confirmed HB vaccine status based on the occupation.

		Total	Dentists	Dental staff	*p* Value
			*n* = 363	*n* = 1032	
HB vaccination	Vaccinated	824 (59.1%)	267 (73.6%)	557 (54.0%)	<.0001
	Unvaccinated	571 (40.9%)	96 (26.4%)	475 (46.0%)	

*Note*: Differences between the two groups were analyzed using Fisher's exact test.

Abbreviation: HB, hepatitis B.

### 3.3 Differences in vaccination status among the 1395 subjects with confirmed HB vaccination status

The 1395 subjects with confirmed HB vaccination status were divided into two groups: vaccinated and unvaccinated. Significant differences were observed between the two groups in terms of age, gender, age group, occupation, HBsAg positivity, and anti‐HBs positivity. The vaccinated group was significantly younger (42.7 ± 14.4 vs. 48.9 ± 15.7; *p* < .0001), had a greater proportion of males (66.0% vs. 34.0%; *p* = .0022), included more dentists (32.4% vs. 16.8%; *p* < .0001), showed lower HBsAg positivity (0.1% vs. 1.2%; *p* < .0097) and had higher anti‐HBs antibody positivity (60.6% vs. 15.4%; *p* < .0001) compared to the unvaccinated group (Table 3).

**Table 3:** Characteristics of 1395 subjects with confirmed HB vaccine status.

Characteristics		Total	Vaccinated	Unvaccinated	*p* Value
		1395	824 (59.1%)	571 (40.9%)	
Age, mean ± standard deviation, years		45.3 ± 15.2	42.7 ± 14.4	48.9 ± 15.7	<.0001
Sex	Male	356	235 (66.0%)	121 (34.0%)	.0022
	Female	1039	589 (56.7%)	450 (48.3%)	
Age, years, *n* (%)	19	1 (0.1%)	0 (0%)	1 (0.2%)	NS
	20−29	300 (21.5%)	207 (25.1%)	93 (16.3%)	<.0001
	30−39	234 (16.8%)	152 (18.5%)	82 (14.4%)	.0491
	40−49	303 (21.7%)	202 (24.5%)	101 (17.7%)	.0024
	50−59	249 (17.8%)	135 (16.4%)	114 (20.0%)	NS
	60−69	224 (16.1%)	99 (12.0%)	125 (21.9%)	<.0001
	70−79	80 (5.7%)	28 (3.4%)	52 (9.1%)	<.0001
	80−89	4 (0.3%)	1 (0.1%)	3 (0.5%)	NS
Occupation	Dentists	363 (26.0%)	267 (32.4%)	96 (16.8%)	<.0001
	Dental staff	1032 (74.0%)	557 (67.6%)	475(83.2%)	
HBsAg, *n* (%)	Positive	8 (0.6%)	1 (0.1%)	7 (1.2%)	.0097
	Negative	1387 (99.4%)	823 (99.9%)	564 (98.8%)	
Anti‐HBs, *n* (%)	Positive	587 (42.1%)	499 (60.6%)	88 (15.4%)	<.0001
	Negative	808 (57.9%)	325 (39.4%)	483 (84.6%)	
Anti‐HCV, *n* (%)	Positive	9 (0.6%)	5 (0.6%)	4 (0.7%)	NS
	Negative	1386 (99.4%)	819 (99.4%)	567 (99.3%)	

*Note*: Differences between the two groups were analyzed using Wilcoxon signed‐rank test and Fisher's exact test.

Abbreviations: anti‐HB, antibody to HBsAg; HB, hepatitis B; HBsAg, hepatitis B surface antigen; HCV, hepatitis C virus; NS, not significant.

The HBsAg and anti‐HBs positivity rates in the unvaccinated group (571 subjects) showed an increasing trend with increasing age (Table 4). Specifically, the HBsAg positivity rate was 1.0% among those in their 40s, 1.8% among those in their 50s, 1.6% among those in their 60s, and 3.8% among those in their 70s. On the other hand, anti‐HBs positive rates were 2.2% for those in their 20s, 2.4% for those in their 30s, 12.9% for those in their 40s, 18.4% for those in their 50s, 21.6% for those in their 60s, 40.4% for those in their 70s, and 66.7% for those in their 80s.

**Table 4:** HBsAg and anti‐HBs positivity by age among 571 unvaccinated subjects.

Age, years, *n*	Unvaccinated, *n*	Positive of HBsAg, *n* (%)	Positive of anti‐HBs, *n* (%)
	*n* = 571	*n* = 7	*n* = 88
19	1	0 (0%)	0 (0%)
20−29	93	0 (0%)	2 (2.2%)
30−39	82	0 (0%)	2 (2.4%)
40−49	101	1 (1.0%)	13 (12.9%)
50−59	114	2 (1.8%)	21 (18.4%)
60−69	125	2 (1.6%)	27 (21.6%)
70−79	52	2 (3.8%)	21 (40.4%)
80−89	3	0 (0%)	2 (66.7%)

Abbreviations: anti‐HB, antibody to HBsAg; HBsAg, hepatitis B surface antigen.

**Figure 1:**
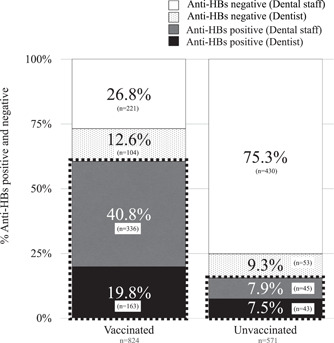
Relationship between the vaccination status and anti‐HBs based on the occupation. The following symbols represent different groups: “black square” denotes dentists with positive anti‐HBs antibodies, “gray square” represents dental staff with positive anti‐HBs antibodies, “dotted square” indicates dentists with negative anti‐HBs antibodies, and “white square” represents dental staff with negative anti‐HBs antibodies. In the vaccinated group (*n* = 824), the positivity rate of anti‐HBs antibodies was 60.6%, while the negativity rate was 39.4%. In the unvaccinated group (*n* = 571), 84.6% tested negative for anti‐HBs antibodies. The proportion of dental staff (*n* = 475) was higher than that of dentists (*n* = 96) in the unvaccinated group, resulting in a higher number of individuals with negative anti‐HBs antibodies among the dental staff (*p* < .0001). anti‐HBs, antibody to HBsAg; HB, hepatitis B.

Figure 1 shows the relationship between the vaccination status and anti‐HBs based on occupation. In the vaccinated group, 60.6% were anti‐HBs positive; among the 39.4% (325/824) subjects with a history of vaccination who were anti‐HBs negative, 12.6% were dentists, and 26.8% were dental staff. Among the 571 subjects without a vaccination history, 53 dentists (9.3%) and 430 dental staff (75.3%) tested negative for anti‐HBs antibodies (Figure 1).

### 3.4 The 11 subjects positive for anti‐HCV antibodies

As shown in Table 5, the 11 subjects positive for anti‐HCV antibodies comprised seven dentists and four dental staff (nine males and two females; average age, 64.4 ± 13.1 years). Titers of anti‐HCV antibody were found for nine of the 11 subjects (two did not provide data); of the nine, the subject with a COI of more than 300 for anti‐HCV antibody titers (No. 3 in Table 5) was most likely to be a persistently HCV infected individual. Regarding the history of antiviral therapy, seven subjects had received treatment, three had not, and one did not respond to the question. The treatment outcomes of the seven subjects who underwent antiviral therapy remain unknown because HCV RNA measurements were not conducted during the health examination.

**Table 5:** The list of 11 subjects positive for anti‐HCV antibodies.

No	Occupation	Sex	Age	Anti‐HCV	Titer of anti‐HCV (COI)	Classification of titer of anti‐HCV	History of antiviral treatment for hepatitis C
1	Dentist	Male	75	Positive	15.16	Middle	Yes
2	Dentist	Male	74	Positive	3.2	Low	Yes
3	Dentist	Male	72	Positive	≥300.00	High	Yes
4	Dentist	Male	72	Positive	2.91	Low	No
5	Dentist	Male	70	Positive	5.9	Low	Yes
6	Dentist	Male	70	Positive	14.7	Middle	Yes
7	Dentist	Male	60	Positive	2.75	Low	No response
8	Dental staff	Male	66	Positive	18.66	Middle	Yes
9	Dental staff	Female	63	Positive	Unknown	NA	No
10	Dental staff	Male	57	Positive	Unknown	NA	Yes
11	Dental staff	Female	29	Positive	2.76	Low	No

*Note*: Classification of titer of anti‐HCV: Low 1.0 to <10.0, middle 10.0 to <200.0, high ≥200.0

Abbreviations: COI, cut‐off index; HCV, hepatitis C virus; NA, not available.

## 4 DISCUSSION

As reported previously, healthcare workers have a higher HBV infection rate than the general adult population (Ciorlia & Zanetta, 2005); similar results were obtained amongst the Dental National Health Insurance Society members in the Oita prefecture in our previous report (Nagao et al., 2021). DHCWs are 10 times more likely to be HBV carriers than the average citizen (Araujo & Andreana, 2002). The factors contributing to a high rate of HBV infection among DHCWs include the elevated risk of needlestick injuries and cuts, as well as the insufficient coverage of the HB vaccination. Exposure to needlestick injuries has been reported to be high, ranging from 70.3% to 73.2% among dentists and 52.6%−77.2% among dental staff in Japan (Kobayashi, 2015; Nagao et al., 2022). The infection rate is reported to be 37%−62% (with the incidence of hepatitis ranging from 22% to 37%) in cases of injuries caused by needles contaminated with blood positive for HBsAg and hepatitis B e antigen (HBeAg). In comparison, the infection rate with blood positive for HBsAg but negative for HBeAg is reported to be 23%−37% (with an incidence of hepatitis ranging from 1% to 6%) (U.S. Public Health Service, 2001; Werner, 1982). On the other hand, the transmission rate of HCV when exposed to HCV‐positive blood varies by country, but it is reported to be approximately 2% (Lanphear et al., 1994; Mitsui et al., 1992; Puro, 1995; Ryoo et al., 2012; U.S. Public Health Service, 2001).

**Figure 2:**
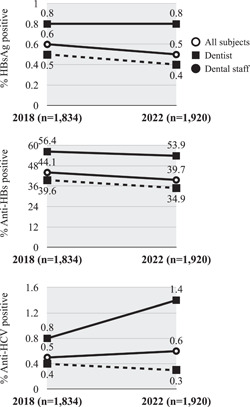
Comparison of hepatitis screening among DHWs in the Oita prefecture between the 2018 study (*n* = 1834) and the current study (2022; *n* = 1920). The figure compares the average positivity rates for HBsAg, anti‐HBs, and anti‐HCV antibodies. The solid circle represents all participants, the solid square represents dentists, and the dashed circle represents dental staff. The positivity rate for anti‐HBs antibodies showed an average decrease of 4.4%; an increase in anti‐HCV antibody positivity was observed among the dentists (from 0.8% to 1.4%). anti‐HBs, antibody to HBsAg.

In the current study, screening for hepatitis B and C virus infection was conducted on 1920 DHCWs in the Oita prefecture to investigate the preventive effect of previous HB vaccinations. The survey results showed that the positivity rates of HBsAg, anti‐HBs, and anti‐HCV were 0.5%, 39.7%, and 0.6%, respectively. Compared to the values for HBsAg, anti‐HBs, and anti‐HCV in the 2018 survey (0.6%, 44.1%, and 0.5%, respectively) (Nagao et al., 2021), the 2022 results did not demonstrate any major change, but the anti‐HBs positivity rate was lower by about 4% (Figure 2). The target populations in the two studies were not identical but belonged to the same district. The causes of the decline in the anti‐HBs positivity may include attenuation of the immune response over time and the difference in the population (especially the HB vaccination status of the DHCWs). Anti‐HBs titers decay over time with the increase in the postvaccination period (Phattraprayoon et al., 2022; Sahana et al., 2017).

In the present study, the vaccination coverage among subjects who had a confirmed history of HB vaccination (excluding those who answered “unknown” or did not provide information) was 59.1%, and the rate of non‐vaccination was 40.9%. Dentists had a significantly higher vaccination rate than the dental staff (73.6% vs. 54.0%, *p* < .0001). In our previous study conducted in the same region in 2021, we focused on hepatitis education and online surveys and reported a HB vaccination rate of 65.8% among dentists and 59.7% among dental staff (Nagao et al., 2022). Note that this previous study included not only dentists but also dental staff (223 dentists and 293 dental staff). Thus, the findings of the current study showed an increased proportion of vaccination among dentists, which may be attributed to past hepatitis educational activities involving educational booklets and the increased awareness about infection prevention due to the COVID‐19 pandemic. As for the lower vaccination rate among dental staff, the difficulty of education dissemination during the COVID‐19 disaster, lack of awareness of the vaccination subsidy system, and access problems to vaccination medical facilities may have contributed to the lower rate. To improve the vaccination rate, it is important to disseminate the vaccine guidelines and provide ongoing hepatitis education.

In the present study, vaccinated individuals were found to be predominantly younger males and dentists, with a low HBsAg positivity rate and a high anti‐HBs positivity rate. On the other hand, among the unvaccinated group, a significant proportion of the dental staff tested negative for anti‐HBs antibodies, indicating the urgent need for preventive vaccination in this group. A trend toward an increased risk of HBV infection with increasing age was also evident in the unvaccinated group. In particular, the HBsAg‐positive rate increased markedly among the elderly, reaching 3.8% among those in their 70s. On the other hand, the anti‐HBs antibody positivity rate also increased significantly among the elderly, reaching 66.7% among those in their 80s. This suggests that elderly dental professionals may be more vulnerable to HBV infection.

A significant challenge remains in the high proportion of unvaccinated DHCWs in Japan. The risk perception of HB among healthcare workers is reported to be correlated with HB vaccination (Mubaraki et al., 2019) and safe practices (Sawadogo et al., 2022). Therefore, continuous efforts are necessary to educate DHCWs about the importance of vaccination to reduce the proportion of unvaccinated individuals. The HB vaccine coverage rate among DHCWs in Japan was reported as 20.6% in the Aichi and Hiroshima prefectures (Inoue et al., 2023), 48.2% in the Fukuoka prefecture (Nagao et al., 2008), 59.4% in Tokyo (Kobayashi, 2015), 60.6% in the Yamaguchi prefecture (Konishi et al., 2007), and 62.4% in the Oita prefecture (Nagao et al., 2022). We previously reported that the HBV infection rate in non‐vaccinated Japanese DHCWs was about 16.9 times higher than that in vaccinated workers (Nagao et al., 2008). Our report showed that 98.5% of DHCWs remained uninfected by HBV after vaccine administration, whereas one in four non‐vaccinated workers had a previous HBV infection (25.4%), characterized by HBsAg‐negative and HB core antibody (anti‐HBc)‐positive serology, indicating a high infection rate (Nagao et al., 2008). Other reports also indicate a fivefold higher HBV infection rate among non‐vaccinated individuals (Cleveland, 1996).

There is a disparity in the HB vaccine coverage rate between dentists and dental staff, highlighting the need to establish workplace environments and educational programs. Developing the workplace environment involves ensuring proper hygiene management and implementing thorough infection prevention measures.

Furthermore, it is essential to monitor the vaccine coverage rates and the status of infection risk regularly. Conducting regular check‐ups and implementing measures to improve infection prevention practices are crucial. These efforts will enhance the vaccine coverage rates and reduce the risk of infection.

In this study, the positive rate of anti‐HCV among dentists was 1.4%, which was higher than the value (0.8%) observed 4 years ago in the 2018 study (Nagao et al., 2021) (Figure 2). Among the 11 anti‐HCV‐positive subjects, the proportions of older individuals and males were high; moreover, seven had a history of antiviral treatment, but the status of viral elimination was unknown. The positive rate of anti‐HCV antibodies increases with age (Moriya et al., 1999), and elderly individuals are at an increased risk of HCC (Asahina et al., 2010). Although the specific factors contributing to the increase in the anti‐HCV positivity rate could not be clearly identified within the provided information, it is important to educate DHCWs about the effectiveness of direct‐acting antiviral agents (DAAs) in eliminating HCV (Mangia et al., 2020) and reducing the incidence of HCC (Ioannou et al., 2018). The DHCWs should be made aware of the long‐term benefits of DAAs (Ioannou & Feld, 2019).

Both patients and DHCWs have the potential to act as hosts for microorganisms. Dental treatment, especially oral surgery, is considered a risk factor for HBV and HCV infections (Averbukh & Wu, 2019; Caminada et al., 2023; Mahboobi et al., 2010, 2013; Redd et al., 2007; Younai, 2010). Improper use of sterile techniques and insufficient medical education have been reported to increase the risk of HCV transmission during dental procedures (Averbukh & Wu, 2019). Furthermore, invasive procedures have been associated with an elevated risk of acquiring acute hepatitis B and C, with a strong correlation between HBV infection and gynecology, otolaryngology, and cardiovascular/thoracic surgery (Caminada et al., 2023). Similarly, strong associations have been reported between HCV infection and neurosurgery, otolaryngology, and vascular surgery interventions (Caminada et al., 2023).

Since 2018, dental clinics in Japan have been required to submit declarations for the facility standards to calculate the consultation fees for insurance‐based dental treatments. Dentists must undergo training every 4 years on infection control measures within the dental outpatient setting. From 2022, additional training on emerging infectious diseases is obligatory for dentists and staff members. However, it should be noted that these facility standards do not guarantee the absolute preparedness of clinical practices, and the healthcare sector necessitates continuous learning and evidence‐based approaches to address the latest developments.

This study has several limitations. First, conducting additional evaluations for individuals who tested positive for HBsAg or anti‐HCV antibodies was not feasible. The participants who received notifications opted for further diagnostic testing based on their judgment, making it impossible to track their outcomes. Additionally, data regarding anti‐HBs titers could not be obtained. Furthermore, we could not acquire information regarding the number and timing of HB vaccination doses.

## 5 CONCLUSION

The overall rate of HB vaccination in this study was 59.1%. Dental staff had a significantly lower rate (54.0%) than dentists (73.6%), resulting in higher anti‐HBs positivity among dentists. Raising awareness about the importance of hepatitis prevention vaccination among dental staff is crucial. The Dental Association should take the lead in providing continuous hepatitis information to DHCWs.

## AUTHOR CONTRIBUTIONS

Yumiko Nagao interpreted data, performed the statistical analysis, and wrote the manuscript. Tetsuya Kimura and Haruhiko Wakita collected data from participants. Kiyohide Tomooka and Haruhiko Wakita interpreted data and added intellectual revision. All authors have read and approved the final version.

## CONFLICT OF INTEREST STATEMENT

The authors declare no conflict of interest.

## Data Availability

All the data sets generated and analyzed in the present study are included in this published article.
